# An Overview of Pterin Analysis in Biological Samples: From Occurrence and Properties to Sample Pretreatment Combined With Hyphenated Separation Techniques

**DOI:** 10.1002/jssc.70483

**Published:** 2026-07-06

**Authors:** Jindřich Brejcha, Zuzana Bosakova

**Affiliations:** ^1^ Department of Philosophy and History of Science Faculty of Science Charles University Prague Czech Republic; ^2^ Department of Analytical Chemistry Faculty of Science Charles University Prague Czech Republic

**Keywords:** biological samples, pretreatment, pterins, separation techniques

## Abstract

Pterins are a structurally diverse group of biologically active compounds within the pteridine family, with key roles in pigmentation, redox metabolism, light sensing, and cellular signaling across a wide range of organisms. Their quantification in biological samples is analytically demanding due to their high polarity, chemical instability, and the presence of multiple oxidation states. This review presents an integrated overview of pterin occurrence, structure, and physicochemical properties, followed by a detailed discussion of sample preparation strategies designed to ensure compound stability and analytical accuracy. Methods such as chemical oxidation, photochemical derivatization, and antioxidant stabilization are evaluated in the context of various biological matrices. We further examine state‐of‐the‐art analytical techniques that combine separation with detection, including capillary electrophoresis, gas and liquid chromatography coupled with fluorescence, UV, electrochemical, or mass spectrometric detection. Particular attention is given to recent advances in LC–MS techniques, including both tandem mass spectrometry (LC–MS/MS) and high‐resolution approaches (e.g., HPLC–Q/TOF‐MS), which have greatly improved the sensitivity, selectivity, and throughput of pterin analysis, especially in combination with HILIC separation mode. These developments support the growing use of pterins as biomarkers in clinical diagnostics and physiological research, and underscore the importance of robust, matrix‐appropriate analytical protocols tailored to the specific challenges posed by this compound class.

## 1 Introduction

Pteridines are a class of heterocyclic compounds, composed of a pyrimidine ring fused to a pyrazine ring, that comprise many derivatives with different physiological functions [1]. There are several groups of pteridines: pterins, lumazines, folates, and, from certain points of view, also flavins. Although structurally related, the different classes of pteridines are typically treated as separate analytical targets. Furthermore, folates constitute a large and well‐established field of research with numerous dedicated reviews addressing their chemistry, biological functions, and analytical determination [2]. The present review therefore focuses on pterins and the analytical approaches used for their determination in biological samples.

**FIGURE 1:**
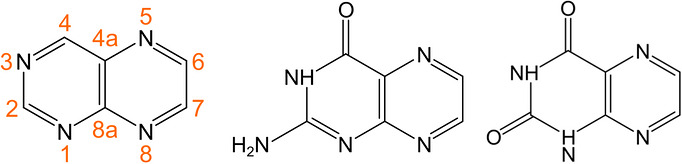
Structures of pteridine (left), pterin (middle), and lumazine (right).

The pterins have an amino group at Position 2 and a keto group at Position 4, while lumazines are 2,4‐dioxopteridines [3]. The pteridine numbering system and structures of pterin and lumazine are illustrated in Figure 1. Folates consist of a pterin linked to a *p*‐aminobenzoyl group by way of a methylene bridge, which is further linked to poly‐glutamate [4]. Compared to the three groups mentioned above, flavins are the most distinct in the sense that they incorporate an additional cycle to the pteridine ring. However, with riboflavin, 6,7‐dimethyl‐8‐ribityl‐lumazine is its immediate precursor during synthesis [5].

Pteridines are structurally similar to purines, but possess a pyrazine ring instead of an imidazole ring, which distinguishes them from purines. Purines include compounds that are essential for life as we know it, such as certain bases of nucleic acids and their derivatives, like adenine and guanine, mono‐, di‐, and triphosphates (ATP, GTP), or associated cyclic phosphates (e.g., cAMP) [6]. Purines and pteridines may share prebiotic self‐catalyzed synthetic paths with pyrimidines [7, 8]. Pteridines can arise in nonenzymatic reactions under specific conditions (warm‐little‐pond) that could simulate conditions on prebiotic Earth [9, 10].

The most common group of pteridines are the pterins. Pterins have many diverse biological functions. Of particular interest is the possibility that they were involved as nucleotide‐like monomers in the primordial RNA [11]. In bacteria, pterins serve a range of functions, including molecular communication [12]; additionally, some synthetic pterins have been shown to inhibit the growth of particular bacterial taxa under experimental conditions [13]. Eukaryotes also use pterins to communicate. Slime mold *Dictiostelium lacteum* uses a pterin derivative as an aggregation signal instead of using cyclic adenosine monophosphate (cAMP) like *D. discoideum* [14]. In plants pterins are important in folate synthesis [15]. In animals, pterins also play diverse physiological functions [16].

Pterins are compounds with properties suited for light‐energy capture and transfer [17]. Using fluorescence techniques, one can demonstrate an energy transfer between UV‐absorbing pterins and flavins [18]. Flavins and pterins are involved in light perception in *Euglena gracilis*, suggesting a function in photosensitivity [19]. In cyanobacteria, pterins and flavins also seem to be a part of the photoreceptor apparatus [20]. Pterins are present in the chloroplasts of higher plants [21], and light, which induces their fluorescence, may generate radicals, causing various types of damage in cells [22].

**FIGURE 2:**
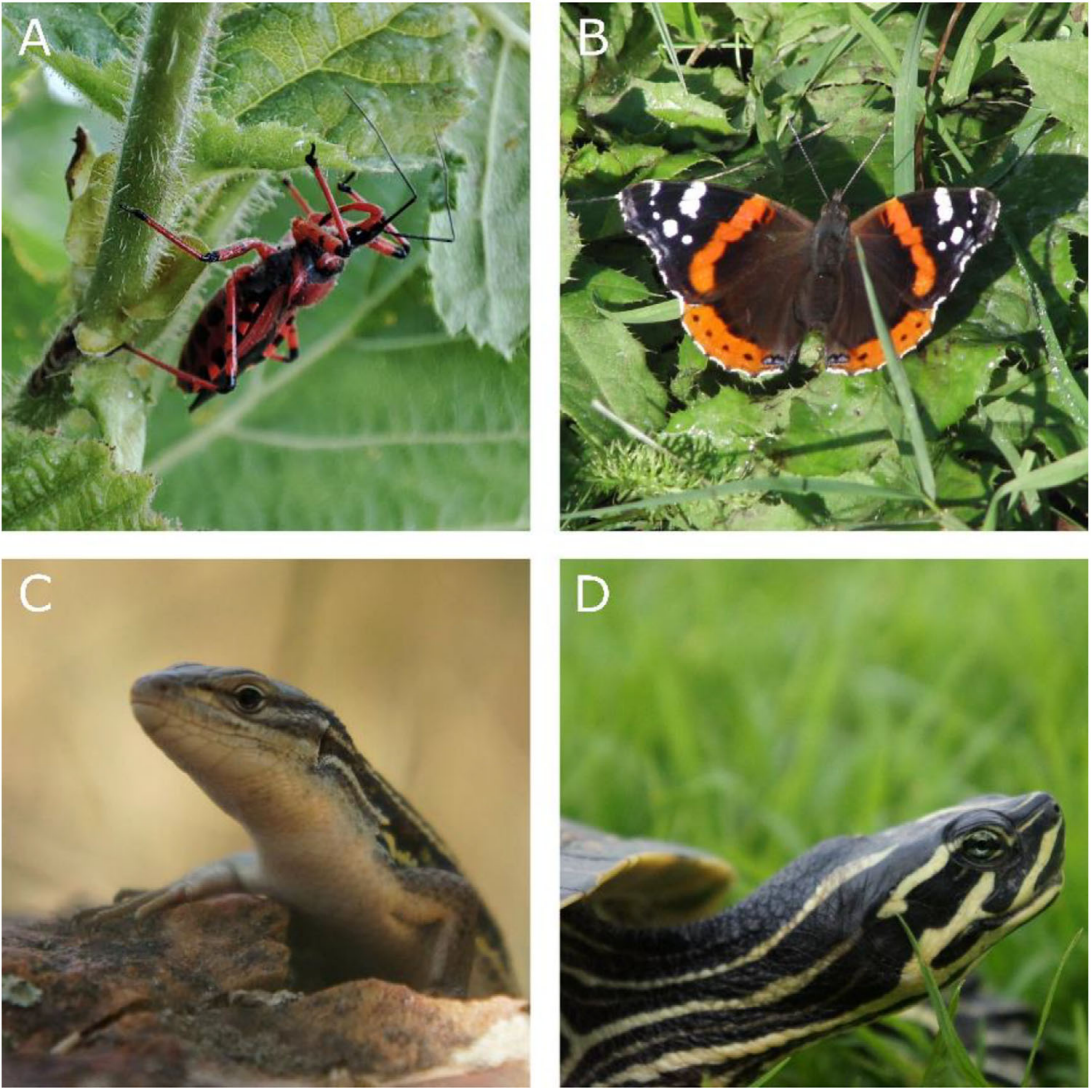
Examples of organisms in which pterin compounds may be expected in the integument. (A) Red‐and‐black assassin bug (*Rhynocoris* sp.), illustrating conspicuous aposematic coloration in insects, (B) red admiral butterfly (*Vanessa atalanta*), a lepidopteran with pigment‐based wing coloration involving pterins and other pigment classes, (C) Algerian sand lizard (*Psammodromus algirus*), representing squamate reptiles with skin coloration produced by chromatophores, where pterins are known or suspected to contribute to color expression, (D) Florida red‐bellied turtle (*Pseudemys nelsoni*), an example of a freshwater turtle with patterned skin coloration in which pterins may occur in the dermal pigment cells.

Perhaps the most prominent function of pterins with respect to light is the production of colors. The first pterin described in nature was one causing the bright colors of butterfly wings [23]. The pterins that produce animal coloration are the most studied in this regard [16, 24]. Interestingly, color‐producing pterins are also present in mushrooms [25]. Typically, pterins absorb certain wavelengths of light, which leaves the rest of the visible light spectrum to be reflected from the tissues, which is perceived as a color. Examples of such filtering effects are the vivid colors of the skins of reptiles [26, 27] the eyes of birds [28], or the exoskeletons of insects [29] (see Figure 2). In other cases, however, the color may arise from the scattering of light by pterin granules or crystals [30, 31].

From uses that go back to the origins of life on Earth, pterins are likely ancient molecules, deeply intertwined with the existence of life itself. Given their ubiquitous presence in living systems, it is no surprise that pterins play important biological roles as inhibitors, enzymes, cofactors in cell metabolism [32] and so on. From an anthropocentric perspective the importance of pterins lies in the fact that they can serve as important medical markers of various physiological processes. The physiological role and diagnostic value of pterins are discussed in numerous papers [33, 34, 35, 36, 37, 38, 39, 40, 41, 42, 43, 44]. For example, urinary neopterin is an excellent nonspecific marker of systemic inflammation [43], while increased concentrations of pterins in biological fluids may be related to cancer, infection, or autoimmune disorder [33]. The therapeutic potential of pteridine derivatives is summarized in [33], hence our ability to efficiently and sensitively detect them takes on a crucial importance. Therefore, we provide here an overview of recommendations on how to analyze pterins using separation methods.

## 2 Chemical Properties of Pterins

**FIGURE 3:**
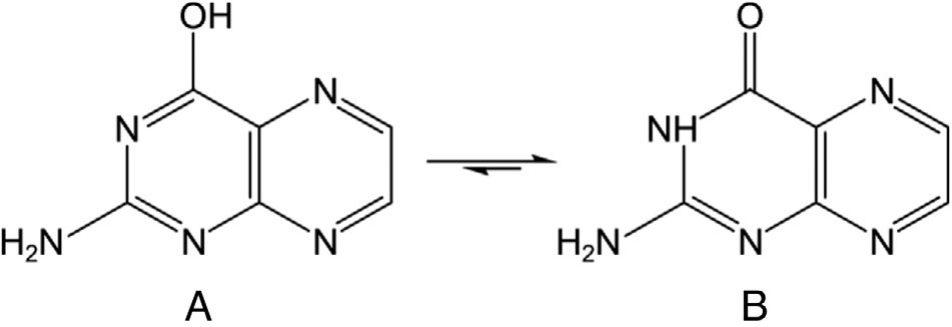
Keto‐enol tautomerism in pterin: (a) enol form (lactim), (b) keto form (lactam).

Pterins exist in two tautomeric forms. Keto‐enol tautomerism in pterins is shown in Figure 3. The keto form prevails due to its higher thermodynamic stability.

**FIGURE 4:**
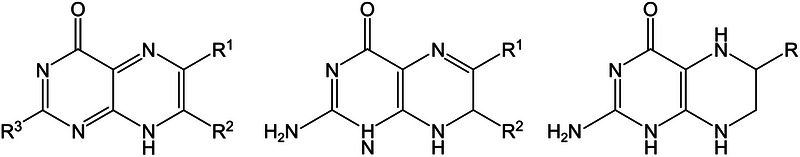
Structure of oxidized pterin (left) and reduced pterins—dihydropterins (middle) and tetrahydropterins (right).

Theoretical results confirm that at least some pterins have the potential to act as electron donors upon excitation by light energy [45]. Participation in redox reactions is one of the fundamental roles of pterins in biology. Pterins can exist in several oxidation states (fully oxidized, semi‐reduced or in a dihydro state and fully reduced or in a tetrahydro state), as indicated by the prefixes tetrahydro‐, trihydro‐ (unstable radicals) or dihydro‐, that specify the number of hydrogen atoms attached to the pyrazine ring. Each oxidation state has a different stability. The fully oxidized state is the most stable. Fully reduced tetrahydropterins are generally unstable in air and oxidized to a dihydro state. Autooxidation of the tetrahydro‐ and dihydro forms with molecular oxygen occurs at neutral pH values [46, 47, 48]. Structures of oxidized and reduced pterins are presented in Figure 4 and in the following Tables 1, 2, 3.

**TABLE 1:** Structure of oxidized pterins, for the backbone, see Figure 4.

Compound	Abbreviation	R1	R2	R3
Pterin	PT	—	—	─NH_2_
Xanthopterin	XP	═O	—	─NH_2_
Isoxanthopterin	ISO	—	═O	─NH_2_
Leucopterin	LEU	═O	═O	NH2
6‐Methylpterin	6‐MP	─CH_3_	—	─NH_2_
6,7‐Dimethylpterin	6,7‐DMP	─CH_3_	─CH_3_	─NH_2_
6‐Hydroxymethylpterin	6‐HMP	─CH_2_─OH	—	─NH_2_
6‐Formylpterin	6‐FP	─CHO	—	─NH_2_
Pterin‐6‐carboxylic acid	PT‐6‐C	─COOH	—	─NH_2_
Biopterin (6‐Biopterin)	BIO 6‐BIO	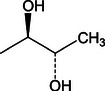	—	─NH_2_
7‐Biopterin	7‐BIO	—	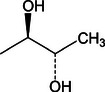	─NH_2_
Neopterin^a^ (6‐Neopterin, D‐Neopterin)	NEO 6‐NEO	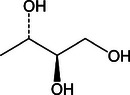	—	─NH_2_
Erythropterin	ERY	═O	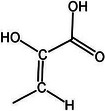	─NH_2_
7‐Neopterin	7‐NEO	—	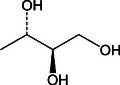	─NH_2_
Oncopterin	ONCO	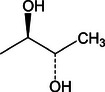	—	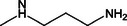

^a^Neopterin (D‐neopterin) has a 1,2,3‐trihydroxypropyl side chain in a 1*S*,2*R* configuration, while D‐monapterin (MON) has a 1*S*,2*S* configuration.

**TABLE 2:** Structure of reduced pterins–dihydropterins, for the backbone, see Figure 4.

Compound	Abbreviation	R1	R2
Dihydrobiopterin	BH2	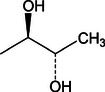	—
Dihydroneopterin	NH2	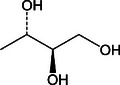	—
Sepiapterin	SP	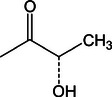	—
7,8‐Dihydroxanthopterin	XH2	═O	—
6‐Methyldihydropterin	6‐MPH2	─CH_3_	—
6‐Hydroxymethyldihydropterin	6‐HMPH2	─ CH_2_─OH	—
6‐Formyl‐7,8‐dihydropterin	6‐FPH2	─CHO	—
7,8‐Dihydro‐6,7‐dimethylpterin	6,7‐DMPH2	─CH_3_	─CH_3_

**TABLE 3:** Structure of tetrahydropterins, for the backbone, see Figure 4.

Compound	Abbreviation	R
Tetrahydrobiopterin	BH4	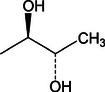
Tetrahydroneopterin	NH4	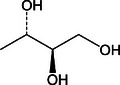

It is known that tetrahydropterins, in particular tetrahydrobiopterin (BH4), are prone to autoxidation in the presence of O_2_. The direct reaction between BH4 and O_2_ is an initiation reaction in which superoxide anion radicals (O_2_^•−^) can be released and rapidly react with BH4 to form intermediate tetrahydrobiopterin radicals (BH4^•+^/BH3^•^) with dihydrobiopterin (BH2) as the end product [47]. The reaction of BH2 or dihydroneopterin (NH2) with the photoinduced singlet oxygen (^1^O_2_) is very fast (including the oxidation of the pterin moiety), yielding biopterin (BIO), neopterin (NEO), and H_2_O_2_ and other products not related to pterins [48]. The different oxidation states of pterins differ in their ability to fluoresce. Fluorescence and photooxidation occur when pterins are excited by electromagnetic radiations at wavelengths between 320 and 400 nm. Absorption spectra and photooxidation depend on the pH and type of substituents [46, 49, 50]. Oxidized pterins (e.g., NEO, BIO, pterin [PT], xanthopterin [XP], and isoxanthopterin [ISO]) show high fluorescence, unlike the tetrahydro‐ and dihydro forms which lack fluorescence. The stability of pterins has been discussed in detail in [44].

Pterins are compounds with high melting points. Unsubstituted pteridines are soluble in water, but common pteridine substituents such as amine, hydroxyl, and keto groups decrease their solubility through their ability to form intermolecular H‐bonds [42]. Due to the presence of weak acidic or basic groups, pterins have acido‐basic properties. In aqueous solutions they behave as weak acids. Their p*K*_a_ lies between 7.3 and 8.3 and is influenced by the type of substituents. Depending on the pH, pterins exist as neutral, positively charged, or negatively charged molecules. Nomenclature of pterins, their biological roles, structure, chemical synthesis, and redox reactivity are discussed in various publications, for example [42, 51].

## 3 Sample Pretreatment

The analysis of pterins is complicated due to the following properties:

- Sensitivity to light and resulting degradation.
- The possibility of diverse oxidation products; reduced pterins do not always oxidize to their fully oxidized parent compound (e.g., BH4 and dihydrobiopterin [BH2] do not always oxidize to biopterin).
- Low solubility, and very low concentrations present in biological fluids.

When selecting a sample preparation method, it is also necessary to consider the type of final analytical method, the complexity of the sample or the need to preserve the original oxidation states [52, 53]. It is difficult to determine all three oxidation forms of pterins in one run. The analysis of the overall amounts of pterins may require either their oxidation or their stabilization by reducing agents. Both approaches have been investigated, and it has been found that the analysis of fully oxidized forms is preferable due to their greater stability [37]. Various oxidation agents have been tested. The most common method is oxidation with iodine/iodide [54, 55, 56]. Iodine oxidation consists of two separate steps. With acidic iodine oxidation, BH4 and BH2 are both oxidized to BIO, whereas NH2 is oxidized to neopterin NEO. With alkaline iodine oxidation, BH4 is oxidized to PT and BH2 to BIO. The amount of BH4 can be calculated from the difference between the amounts of BIO produced through acidic and alkaline oxidation. Using the iodine/iodide method, the oxidation to BIO results in an 80% conversion to PT. A disadvantage of the method is that it can only measure the two oxidized forms combined and does not distinguish BH2 from BIO, [56] (for all the structures, see Tables 1, 2, 3).

Pterin oxidation has also been performed with potassium permanganate in a neutral medium. Durán‐Merás compared the oxidation with iodine/iodide with the KMnO_4_ oxidation method. When using permanganate, he observed no XP signal probably due to a break in the condensed rings causing a subsequent loss of fluorescence. A long oxidation time can also break the pteridine rings, diminishing the fluorescence signal. It can be concluded that the oxidation process greatly affects the proportion of pterins formed [57]. It was shown that PT/creatinine (CREA) and NEO/BIO ratios strongly depend on the time of oxidation and oxidant concentration. The best oxidation yield was achieved after 10 min using KMnO_4_ oxidation and after 40 min using iodide/iodine. Using an alkaline iodine/iodide medium results in only partial formation of BIO from BH4/BH2 because most of the BH4 is oxidized to PT. In a neutral permanganate medium, the oxidation of BH4/BH2 generates mainly BIO and, consequently, the NEO/BIO and PT/CREA ratios decrease significantly. This should be kept in mind when using the NEO/BIO ratio as a clinical criterion [58].

Another oxidation medium is MnO_2_ under an acidic pH which was used for the urine sample preparation in the determination of PT, ISO, BIO, 7‐BIO, NEO, 7‐NEO in urine [59].

An alternative to chemical oxidation is UV photoirradiation of pterins [60, 61]. A post‐column photoderivatization can be carried out with an on‐line photoreactor located between the diode array detector (DAD) and the fast‐scanning fluorescence detector. NEO, BIO, PT, and BH2 can be determined by measuring native fluorescence, using the photoreactor in off‐mode, while BH4 can be determined by measuring the induced fluorescence of the generated photoproducts, using the photoreactor in on‐mode. This protocol has some advantages over the traditional iodine oxidation method which can only determine BH4 and BIO_total_, since the photoderivatization method can also determine BIO, NEO, and PT_initial_, BH2, BH4, and BIO_total_ in the same urine sample. Also, the analysis time per sample, 90 min, is shorter than when applying the classical iodine oxidation method.

Derivatization of pterins is still another option. Derivatization of BH4 with benzoyl chloride followed by LC–MS has been proposed [62]. BH4 was directly derivatized with benzoyl chloride and analyzed by MS/MS. The method is based on the secondary amine group of BH4 at Position 5 reacting with benzoyl chloride. The product of the reaction has very good stability and high MS sensitivity.

An alternative approach to the oxidation of unstable pterins is the stabilization of their reduced forms (see e.g., [49, 63]). Various antioxidants have been tested to avoid degradation of pterins: dithiothreitol (DTT), dithioerythreitol (DTE), and ascorbic acid (AA) [44, 63]. The stability of pterin standards NEO, BIO, NH2, and BH2 with and without the stabilizing agent DTT was studied under varying pH in the range of 3.8–9.8. BH2 proved to have a much greater stability at pH 3.8 than NH2. The decrease of NH2 was lower (∼10%) at a basic pH than at acidic conditions (∼35%). The highest stability for both NH2 and BH2 was obtained at pH 6.8 (the decrease was about 1%). NEO and BIO were stable at acidic (loss was < 2%) and basic (< 5%) pH, but decreased at a neutral pH (< 11%). Different concentrations of DTT (1%–10%) had similar effect on the stability of their dihydroforms. Therefore, 1% DTT is recommended in order to prevent pterin degradation. Lyudnikova et al. [46] observed no oxidation of BH4 during 20 min in acidic solutions at pH < 3.0, but it oxidized rapidly at pH values above 3.0. Also, NH2 was cleaved to 7,8‐dihydroxanthopterin (XH2) at neutral pH values while NH2 to NEO oxidized at acidic conditions [64]. At pH 7.4 in aqueous solutions, BH4 was oxidized by dissolved molecular oxygen to H_2_O_2_ as a major end product. AA prevents the auto‐oxidation of BH4 and 3 mmol L^−1^ AA almost completely stabilizes 25 µmol L^−1^ BH4 [65].

The effect of DTT on the stability of urine samples containing reduced (NH2, BH2, and BH4) and oxidized (NEO, BIO) forms at room temperature has been tested. In general, oxidized and dihydro forms are stable at 0.1% DTT in acetonitrile over 72 h. BH4 is less stable, as evidenced by a significant decrease in its content after 72 h. BH4 is most stable up to 24 h, when the decrease in its content is ∼2%. Therefore, the analysis of one batch should be completed within 24 h to prevent degradation of BH4 [66]. Reliable determination of BH4 and BIO_total_ content in plasma requires that 0.1% DTT be present in blood tubes for at least 2–3 h before centrifugation [63].

Biondi et al. [67] studied BH4 autoxidation, oxidation by H_2_O_2_, that induced by superoxide and by hydroxyl radicals generated via the Fenton reaction. Oxygen‐induced BH4 autoxidation and superoxide‐catalyzed oxidation produce predominantly BH2, while hydroxyl radical‐driven BH4 oxidation produces mainly PT and XH2.

*Urine sample* preparation is simple; it consists in the dilution, centrifugation, and filtration [66, 68] to remove proteins which could interfere with the HPLC analysis. To increase selectivity of the pretreatment, solid‐phase extraction is recommended [69]. Three SPE sorbents including hydrophilic–lipophilic‐balanced polymer phase Oasis HLB, porous graphitic carbon HyperSep Hypercarb (PGC), and mixed‐mode (reversed‐phase and cation‐exchange) sorbent DSC‐MCAX were tested and DSC‐MCAX produced optimal results [69].

*Cerebrospinal fluid* (CSF) samples contain fewer interfering compounds compared to other biological matrices. Only dilution and filtration steps are required before HPLC analysis [70]. Pterins in CSF are present in the low nmol L^−1^ range. To prevent auto‐oxidation of pterins, DTT and diethylenetriaminepentaacetic acid (DETAPAC) are added to fresh samples and immediately frozen and stored at −80°C until analysis [71, 72]. If the sample is contaminated by blood, it should be immediately centrifuged to remove red blood cells which cause oxidation of amine‐bearing metabolites [71]. Guibal et al. [73, 74] studied the stability of pterins in CSF samples frozen at −80°C immediately after collection. They detected no significant change in pterin concentration after 24 months of storage. The addition of a preservative agent to the samples can thus be avoided.

*Dry blood spot* (DBS) Selective screening for BH4 deficiency, which is mandatory for every newborn with even slightly elevated blood phenylalanine levels, is currently based on the analysis of NEO_total_ and BIO_total_ in urine [75]. The measurement of NEO, BIO, and PT in DBS has been developed as an alternative to urine samples. DBS samples collection, handling, preservation, and conservation procedures are more convenient than for urine analyses, therefore, the detection of NEO and BIO in DBS samples offers clear advantages for the diagnosis and therapeutic monitoring of individuals affected by these disorders. Furthermore, the assessment of pterins in DBS could, if proved to be reliable, improve the early identification of BH4 disorders when used as a second‐line test in newborns with hyperphenylalaninemia (HPA, defects in synthesis or regeneration of BH4, BH4 deficiency). Pterins in DBS which are extracted with diluted HCl, sonicated and ultrafiltrated before HPLC analysis [76, 77] demonstrated the stability during storage at room temperature in the dark for a period of up to 16 days and at −20°C after 6 and 18 months.

*Plasma* pretreatment involves protein precipitation (e.g., with trichloroacetic acid or acetonitrile) [63, 78, 79]. Acid precipitation removes or co‐precipitates varying amounts of pterins which results in an under‐estimation of NH4 and NEO_total_ levels in plasma [78]. This problem can be overcome by using acetonitrile for precipitation. The procedure also allows an accurate determination of plasma NH2, which is not oxidized to NEO. For a reliable determination of BH4 and BIO_total_ in plasma, blood tubes must contain DTT and the time between blood collection and centrifugation is critical [63]. Tonero [79] applied precipitation of the serum proteins with trichloroacetic acid, centrifugation, and clean‐up with an Isolute ENV+ (hydroxylated polystyrene‐divinylbenzene copolymer) cartridge. The application of the cartridge is necessary if the separation is carried out on superficial particles which strongly retain the serum components and affect the column efficiency.

While pterins are predominantly determined in biological fluids, papers dealing with their analysis in other biological materials have also been published.

*Tissue* analysis usually involves lysis, sonification and deproteinization [80]. The quantification of BH4, BH2, and BIO from freshly prepared human umbilical vein endothelial cells (HUVECs) involves the precipitation of proteins and other cellular components with trichloroacetic acid. Antioxidants, AA and DTT are usually also added to stabilize redox conditions in the lysates [81]. The cells are separated by trypsination, centrifuged, and frozen. Human breast milk is treated similarly: precipitated, centrifuged and the supernatant used immediately for analysis. A simple and rapid LC–MS/MS method has been developed for the quantification of BH4 and dopamine in rat brain. Proteins in the samples are precipitated with acetonitrile and then the supernatant is separated. DTT is added to prevent the oxidation of the pterins in the sample [82].

For extraction of pterins from *animal material*, alkaline or acid extraction were usually used [83, 84, 85]. The high pH conditions often lead to decomposition of pterins [86]. A new extraction method based on dimethyl sulfoxide (DMSO) was used to extract pterins contained in various biological tissues (integument, skin, and eye). This extraction is advantageous in that it extracts pterins in their original form without causing tissue decomposition (integument, skin, and eyes), so that direct collection of DMSO from the sample is sufficient and the organic extract can be analyzed immediately [26, 27, 29, 87, 88].

## 4 Separation and Detection Methods

### 4.1 Gas Chromatography

Gas chromatography (GC) can only be used for the analyses of volatile and thermally stable compounds. GC was used in the early stages of development of methods for pterin analysis [89, 90, 91, 92, 93]. GC with mass spectrometric detection is more sensitive than liquid chromatography (LC) with fluorescence or diode array detections but has several disadvantages. It requires derivatization of polar pterins, sample preparation is more complicated, and the time taken for analysis is usually longer in GC than in LC. GC–MS is therefore less often applied for routine clinical analysis and is mostly used as a complementary technique for confirmation of the structure of pterin derivatives. Trimethylsilyl derivatives of pterins together with methylene units provide reliable identification of these compounds in biological samples and elucidation of the structure of unknown pterins [92]. Recently, ISO was determined in urine together with other metabolites by GC–MS after derivatization with *N*,*O*‐bis(trimethylsilyl)trifluoroacetamide (BSTFA) in 1% trimethylchlorosilane [93].

### 4.2 Capillary Electrophoresis

Capillary electrophoresis (CE) is a useful alternative to HPLC for the analysis of pterins due to its high separation efficiency, versatility, high speed of analysis, small sample requirements, and low consumption of solvents and background electrolytes. The main drawback is the fluctuation in the electroosmotic flow rate, which may be caused by the adsorption of matrix components or even analytes onto the inner wall of the capillary. This leads to lower reproducibility of the electromigration times of analytes.

**FIGURE 5:**
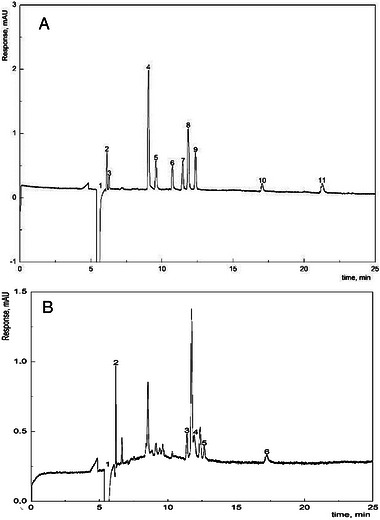
Electropherograms of the separation of (A) 10 pterin standard derivatives in the mixture at the pterin derivative concentration of 2.5 µg·mL^−1^, (1) DMSO (2) SP, (3) XH2, (4) BIO, (5) NEO, (6) PT, (7) ISO, (8) LEU, (9) XP, (10) ERY, and (11) PT‐6‐C. (B) An extract of the cuticle of *Scantius aegyptius* (Pyrrhocoridae), (1) DMSO, (2) SP, (3) ISO, (4) LEU, (5) XP, and (6) ERY. BGE consisting of 2 mmol·L^−1^ Na_2_EDTA, 100 mmol·L^−1^ Tris, and 100 mmol·L^−1^ boric acid, pH 9.0. Electrokinetic injection for 10 s at 20 kV, separation at 20 kV, temperature 30°C and UV detection at 250 nm.  [29].

The pterins were separated well using a basic electrolyte consisting of 0.1 mol L^−1^ Tris‐0.1 mol L^−1^ borate‐2 mmol L^−1^ EDTA. Their separation is very pH sensitive [29, 94, 95, 96]. UV detection at 250 nm successfully permits the identification and the quantification of the relative abundance of 10 pterin derivatives, sepiapterin (SP), XH2, BIO, NEO, PT, ISO, leucopterin (LEU), XP, erythropterin (ERY) and pterin‐6‐carboxylic acid (PT‐6‐C), responsible for the warning coloration in some Heteroptera. All pterins are well separated at pH 9.0 (see Figure 5). Limits of detection (LOD) are in the range of 0.04–0.99 µg mL^−1^ [29].

UV detection is not sensitive enough to permit the detection of the minute amounts of pterins in clinical samples [94]. Laser‐induced fluorescence (LIF) is a method of detection that is extremely sensitive (about 1000 times more than traditional UV detection) and it can be applied as pterins exhibit native fluorescence. CE with an in‐house LIF detector has been developed for determination of pterins in urine samples [95, 96]. The detection limits were in the range of 10^−10^–10^−14^ mol L^−1^. Good separations of urine pterins was obtained only within a narrow range of pH 8.6–8.8. The separation of NEO and 6‐hydroxymethypterin (6‐HMP), the most important pterins measured in urine, is especially sensitive to pH fluctuations due to their similar molecular structures. It was also noted that fluctuations in temperature during the experiments deteriorate the separation [96].

Gamagedara et al. [97] analyzed pterins in urine samples from cancer patients and healthy subjects by HPCE‐LIF as well. They studied eight pterins and obtained LODs for each pterin at the level of 2.5·10^−10^ mol L^−1^ except for PT whose LOD was 4.72·10^−10^ mol L^−1^. HPCE‐LIF was proved to be a fast, simple, and sensitive method. Ma et al. [98] reviewed methods useful for the determination of urinary cancer biomarkers including pterins by CE.

Grochocki et al. [99] evaluated an alternative, cheaper detector, a light‐emitting diode induced fluorescence (LEDIF) detector, for the determination of pterins in urine samples after CE separation. The sensitivity of CE‐LEDIF is about two orders of magnitude lower than that of the CE–LIF technique, and it was concluded that the lower sensitivity is a consequence of employing a less powerful light source as well as a mismatch of the wavelengths used. The method has been validated and applied to the analysis of urine samples from healthy individuals and cancer patients. It was noted that higher sensitivities could be achieved using on‐line preconcentration techniques, and that more sophisticated instruments, such as HPCE–MS and LC–MS/MS, should be used to verify the potential of pterins as cancer biomarkers.

### 4.3 Liquid Chromatography

LC is the most commonly used method for the analysis of pterins. Various stationary and mobile phases and detection modes have been evaluated [53, 67, 79, 100, 101, 102, 103, 104, 105, 106, 107], but reversed‐phase LC is the most widely used for quantitative analysis. Typical mobile phases containing small amounts of organic modifiers together with gradient elution are used to achieve good separations, as pterins are relatively polar molecules. Biondi et al. [67] carried out HPLC analysis of BH4 and its main metabolites, BH2, BIO, and PT, using a Waters Atlantis dC‐18 5 µm reverse phase column. Isocratic elution with a multicomponent mobile phase consisted of NaH_2_P0_4_, citric acid, and acetonitrile with octyl sulfate sodium salt as the ion‐pairing agent, including diethylenetriaminepentaacetic acid (DETPA), which chelates transition metals to prevent oxidation of the analytes, and DTT, as a reductant to further stabilize the reduced forms of the pterins, was developed for the separation and resolution of the positively charged analytes. An HPLC method has been reported for the analysis of nine pterins in human serum. Two analytical columns, one C18 with porous particles, the other with fused core particles were compared. The fused core particle column allows adequate separation in one run in 15 min [79].

Hydrophilic‐interaction liquid chromatography (HILIC) is an alternative technique for the separation of polar compounds. In HILIC, analytes are retained in order of increasing hydrophilicity (the opposite order compared to reversed‐phase separations). The HILIC approach is highly advantageous for the analysis of polar pterins as good retentions are achieved using simple volatile mobile phases without any inorganic buffers. This system is also easily compatible with MS detection and allows high sensitivity and selectivity [59, 66, 73, 74, 88, 102, 103, 104, 105, 106].

**FIGURE 6:**
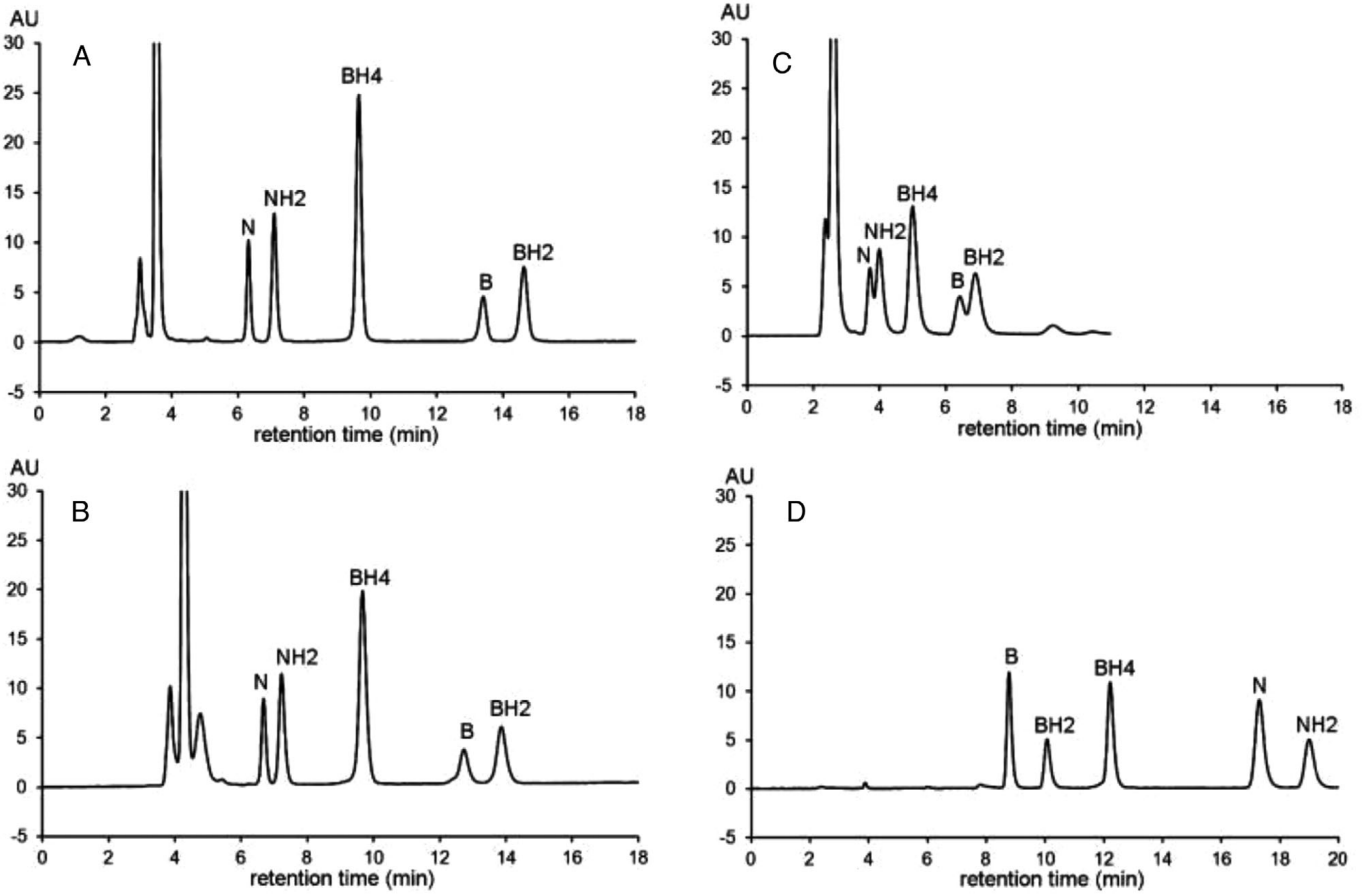
Pterin separation as a function of stationary phase and pH. (a) Atlantis dC18; (b) Eclipse XDB C18; (c) XTerra; (d) ZIC‐HILIC, N‐NEO, B‐BIO. Mobile phase for panels (a–c): pH 7.4, 0.05 M sodium citrate/methanol (97/3, v/v). Mobile phase for panel (d): pH 7.4, 0.2 M ammonium format/acetonitrile (20/80, v/v), flow rate 0.6 mL min^−1^ at 30°C and UV detection at 260 nm for all columns [73].

Giubal et al. developed an HPLC method for the simultaneous determination of all forms of biopterin (BH4, BH2, and BIO) and neopterin (NH2, NEO) in CSF [73]. The use of a phase with bound polar groups (Atlantis) allows for separation of the target pterins without using an ion‐pairing reagent (see Figure 6). Post‐column coulometric oxidation of the reduced forms of pterins enables their direct determination by fluorescence detection.

Allegri et al. [59] achieved the best separation of 6‐ and 7‐positional isomers of BIO and NEO under isocratic mode using an amino‐phase (column LUNA amino [2 mm × 150 mm, 3 µm; Phenomenex]) helped by the presence of the aliphatic polyhydroxylated side chain, which can serve as a retention/discrimination site during the elution process. The stability of most amino columns can be problematic because the amino bonding easily releases from the silica gel. However, Luna NH2 columns exhibit good stationary‐phase stability in both normal‐phase and reverse‐phase modes and over a pH range of 1.5–11.0.

**FIGURE 7:**
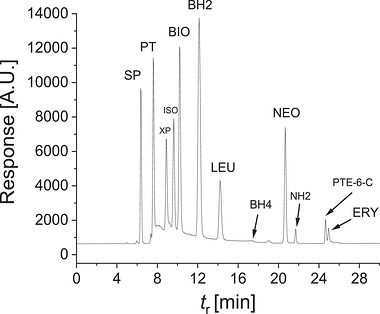
TIC chromatogram of a mixture of 12 pterins, ZIC‐HILIC column, mobile phase: ACN(A)/25 mmol L^−1^ ammonium acetate pH 3/(B) gradient elution: 84% A (14 min), 84%–65% A (6 min), 65% A (6 min), flow rate 0.5 mL min^−1,^ AU—arbitrary units, taken from [105].

HILIC with tandem mass spectrometric detection has been developed for the analysis of pterins, namely for BIO, ISO, LEU, NEO, XAN, and ERY in the cuticle of heteropteran insects [88]. Two columns, Atlantis HILIC Silica (Atlantis HILIC Silica (4.6 mm × 150 mm, 3 µm), based on silica gel and ZIC‐HILIC (4.6 mm × 150 mm, 3.5 µm), based on zwitterionic sulfobetaine groups, were tested. The optimal conditions for the separation of pterins consisted of a ZIC‐HILIC column, a mobile phase composed of acetonitrile/5 mmol L^−1^ ammonium acetate, pH 6.80, 85/15 (v/v), flow rate 0.5 mL min^−1^ and column temperature 30°C. The analysis shows that different species of *Graphosoma* sp. show different abundances of individual pterins, affecting their coloration. By slightly adjusting the separation conditions and using gradient elution, the separation of 12 pterins, including their reduced forms, can be achieved (see Figure 7) [105].

Koslinski et al. [102] compared HILIC Luna cross‐linked diol phase, Li‐Chrospher C‐8 RP phase and Aquasil C‐18 RP stationary phase with hydrophilic endcapping for analysis of 6,7‐DMP, PT, BIO, ISO, NEO, XP, and PT‐6‐C. They achieved the best separations with a C‐8 stationary phase and a mobile phase consisting of methanol in combination with a phosphate buffer at pH 7.0. Nováková et al. [103] tested two HILIC columns (BEH HILIC and BEH Amide) for the group of polar basic pterins (NEO, BIO, NH2, and BH2). While the BEH HILIC column did not completely resolve the NEO–NH2 and BIO–BH2 pairs, the BEH Amide column provided sufficient retention and selectivity for the separation of four pterin derivatives. A HILIC method with an aminopropyl hydrophilic interaction column and fluorescence detection has also been developed for analysis of NEO, BIO, and ISO in urine samples [104].

Retention characteristics of five HILIC columns, containing neutral and possibly negatively charged support (silica, diol, and amide), cationic phase (triazole), and zwitterionic phase (sulfobetaine) were evaluated for the separation of 12 polar pteridines [106]. For silica, diol, amide, and sulfobetaine phases, hydrophilic partitioning mainly contributes to the retention, while electrostatic interactions and hydrogen‐bonding should be considered to understand the elution orders for the triazole phase. The zwitterionic phase (ZIC‐HILIC) provided a stronger retention for all pteridines than the other tested columns.

Various detection methods have been tested: UV, fluorescence, electrochemical, and mass spectrometry (LC–MS, LC–MS/MS). Fluorimetric detection remains the most common technique in the LC analysis of pterins. It is extremely sensitive, but its usefulness is largely limited to oxidized pterins. The reduced pterins show only weak fluorescence and must be converted to their oxidized form prior to analysis. A disadvantage of fluorimetric detection is sensitivity to interference from other fluorescent species and a requirement for standard pterin derivatives. An off‐line photoirradiation LC fluorimetric method to determine BH4 by photogeneration of BIO was described as an alternative method for the chemical oxidation procedure [60]. Fluorimetric detection was coupled with on‐line photochemical oxidation for simultaneous analysis of marker pterins and biopterin reduced forms in urine [61]. A photoreactor was placed between the DAD and the fast‐scanning fluorescence detector. NEO, BIO, PT, and BH2 can be determined by measuring native fluorescence with the photoreactor in off‐mode, and BH4 by the induced fluorescence of the generated photoproducts, with the photoreactor in on‐mode. The DAD is used to determine the reference substance CREA. A Zorbax Eclipse XDB‐C18 column was used for the chromatographic separation, using a 98/2 (v/v), citrate buffer (pH 5.5)/acetonitrile mobile phase, in isocratic mode. Detection limits were 0.2, 13.0, 0.3, 0.3, and 3.5 ng mL^−1^, for NEO, BH2, BIO, PT, and BH4 respectively, and 0.4 µg mL^−1^ for CREA. They presented a comparison of this procedure with the classical iodine oxidation method.

Tornero et al. [79] applied fluorimetric detection to the analysis of pterins in human serum, using excitation at 272 nm by measuring the fluorescence at 410 nm for ISO, at 465 nm for XP, and at 445 nm for the analysis of the other pterins. Detection limits were between 0.07 and 0.61 ng mL^−1^. A combination of fluorimetric and electrochemical detection was applied to the study of the oxidation of BH4 and its main metabolites, BH2, BIO, PT, and XH2. BIO and PT are naturally fluorescent, BH2 was converted to the fluorescent form by post‐column electrochemical oxidation and BH4 and XH2 were detected directly with an electrochemical detector using an electrode set to +450 mV. The LOD for XH2, BIO, PT, BH2, and BH4 were 120, 80, 160, 100, and 60 fmol, respectively [67].

Quadrupole tandem mass spectrometry techniques (e.g., LC–MS/MS) offer sensitivity comparable to that of fluorescence detection without requiring complete separation of pterins and are useful in targeted analysis. A disadvantage is that the suppression of ionization in ESI mode, related to the eluent matrix, cannot be predicted or controlled when analyzing various biological samples due to the sensitivity toward charge competition in the source. The most practical solution is the use of stable isotope‐labeled internal standards. However, their availability for pterin derivatives is very limited. An alternative is to use structurally similar pterins that have no or very low endogenous concentrations such as 6‐MP. If high mass resolution is required for reliable monitoring of pterins without the use of chemical standards, instruments such as Orbitrap‐MS and QTOF‐MS can be used which provide high resolution/accurate mass‐MS spectra with precise mass values, usable for compound identification based on mass spectra with a spectral library [53, 66, 68, 71, 76, 81, 82, 85, 88, 105, 106, 107].

Xiong et al. [66] used a ZIC‐HILIC column with gradient elution for the simultaneous analysis of 12 pterins including oxidized, di‐ and tetrahydroforms in human urine, using DTT as the stabilizing agent, without oxidative pretreatments. The run time of the HPLC–MS/MS analysis was 20 min, and they evaluated the matrix effect for several dilutions of urine. Spiked recovery studies demonstrated that the developed method was accurate (83.1–116.7) and precise (RSD 1.4–15.6). Burton and Ma [34, 68] reviewed the application of the HPLC‐MS technique for separation and quantitation of urinary pteridines as disease biomarkers. Arning and Bottiglieri [71] published the LC–MS/MS method for the determination of BH4, BH2, NEO, and SP in CSF, which utilizes labeled stable isotopes as internal standards. The method is fast and linear over a working range of 3–200 nmol L^−1^.

Ultra‐high‐performance liquid chromatography with tandem mass detection (UHPLC‐ESI‐MS/MS) has also been used for the determination of BIO and NEO in DBS. The developed method enables the evaluation of NEO and BIO in DBS, which are important in newborn screening of HPA. The method is reliable and sensitive and may be proposed as a second test for newborns in addition to monitoring NEO and BIO urine excretion [76]. A simple and rapid LC‐MS/MS method with a Poroshell 120 SB‐C18 column and stepwise gradient was developed for quantitation of BH4, BH2, and BIO in human vein endothelial cells [81]. Total chromatographic run time was 23 min and limits of quantitation were 1 nmol L^−1^ for BH4 and BH2 and 2.5 nmol L^−1^ for BIO. This method provides the simultaneous and direct quantitation of BH4, BH2, and BIO in a single injection. Giron et al. [101] applied a LC–MS method for the determination of pterins NEO, BIO, PT‐6‐C, PT, MON, ISO, XP, 6‐HMP, NH2, BH2, and 6‐MP in their native state. LOD in SIM mode were in the range from the lowest values for PT (from 1.7 to 3.88 ng mL^−1^) to the highest values for XP (10.5–49.9 ng mL^−1^) for healthy volunteers.

High‐performance liquid chromatography–quadrupole time‐of‐flight mass spectrometry (HPLC–Q/TOF‐MS) provides adequate sensitivity, greatly improved selectivity, and the ability to perform MS/MS quantification of pterins. The HPLC–Q/TOF‐MS method was developed to simultaneously quantify seven intracellular pterins and monitor 18 additional, naturally occurring intracellular pterins. The method appears to be highly sensitive (detection limits from 0.1 to 3 µg L^−1^). The detection limits were comparable to previously reported pterin detection limits with fluorescence detectors and mass spectrometry techniques. The HPLC–Q/TOF‐MS technique is both accurate and precise and comparable to other pterin analyses from biological samples [107]. Dilute‐and shoot HPLC‐Q/TOF‐MS method was used for pterin profiling in human urine. The method offers excellent linearity, sensitivity and precision, allowing for simple filtration of urine prior to chromatographic analyses. A total of 135 urine samples from a group of healthy patients and a group of patients with diabetes, hypertension, dyslipidemia, or inflammatory bowel disease were analyzed and the pterin profile was related to fecal calprotectin levels. Pterin levels were compared between both groups, and significant differences were found in the content of some pterins [108]. Table 4 summarizes recently published analytical methods for the LC separation of pterins.

**TABLE 4:** A summary of LC methods published for the analysis of pterins.

Analytes	Biological matrix	Sample preparation	Analytical method/column	LOQ (ng mL^−1^)	Reference
BH4	—	AA as antioxidant, oxidation with O_2_, H_2_O_2_	UV at 305 nm	—	[65]
NEO, BIO, ISO, XP, PT, PT6C, 7‐BIO	Human urine	Iodide/iodine oxidation, KMnO_4_ oxidation, dilution	RPLC‐FD/Zorbax Eclipe XDB‐C_18_	*0.2–4.4	[58]
NEO, BIO, PT, ISO, XP, PT6C, 6,7‐DMP, 6‐HMP	Human urine	Iodide/iodine oxidation, KMnO_4_ oxidation, dilution	RPLC‐FD/Lichrospher C8	0.12–8.7	[102]
NEO, BIO, 7‐BIO, ISO, XP, PT6C, PT, MON, 6‐HMP	Human serum	Iodide/iodine oxidation, Isolute ENV+ SPE	RPLC‐FD/Poroshell 120	0.15–2.93	[79]
NEO, BIO, BH2, PT, BH4	Human urine	Induced fluorescence of BH4 by photoreactor	RPLC‐FD/Zorbax Eclipse XDB‐C18	*0.2–13.0	[61]
BH4, BH2, BIO, NH2	Cerebrospinal fluid	Post‐column coulometric oxidation	RPLC‐FD/Atlantis C18/ZIC‐HILIC	—	[73]
ONCO, NEO, BIO	Human urine	SPE	RPLC‐FD/Purospher RP‐18	—	[69]
BIO, BH4	Human plasma	DTT, DTE, AA as antioxidants	RPLC‐FD/Hypersil C18	*0.2 nmol L^−1^ for BIO	[63]
NEO, BIO, NH2, BH2	Human urine	DTT as antioxidant, DSC‐MCAX SPE	HILIC‐FD/BEH amide	0.5–10	[52]
NEO, NH2, PT, BIO, BH2, XP, ISO, PT6C, MON, 6‐HMP	Human urine	Dilution	RPLC‐MS/Zorbax Eclipse XDB‐C18	5.59–14.0	[101]
NEO, BIO, XP, ISO, LEU, ERY	Cuticle of heteropteran insect species	Extracted by dimethyl sulfoxide	HILIC‐MS/MS/ZIC‐HILIC	0.3–19.3	[88]
PT, ISO, BIO, 7‐BIO, NEO,7‐NEO	Human urine	MnO_2_ under acidic pH	HILIC‐MS/MS/Luna amino	0.021–1.08	[59]
BH4	Human plasma	DTT as antioxidant, derivatization with benzoyl chloride	LC‐MS/MS/QTRAP/Polar‐Imidazole	0.02	[62]
NEO, NH2, PT, XP, ISO, SP, PT6C, 6,7‐DMP, BIO, BH2, BH4, DMH4PT	Human urine	Dilution	HILIC‐MS/MS/ZIC‐HILIC	0.4–40	[66]
NEO, BIO, PT, XP, ISO, PT, PT6C, 6,7‐DMP	Cell	Dilution	RPLC‐QTOF‐ MS/Phenyl‐hexyl	0.1–1.0	[107]
NEO, BIO, PT, XP, ISO, SP, 6,7‐DMP, PT6C, MON, 6‐MP, 6‐HMP, XH2	Human urine	Dilution	RPLC‐MS/MS/Luna Phenyl‐hexyl	0.05–0.3	[68]
NH2, BH2, 6,7‐DMP, MON, NEO, BIO, LEU, PT, XH2, 6‐HMP	Human urine	Filtration	RPLC‐QTOF‐MS/Discovery RP Amide C16	0.54–30	[108]

Abbreviations: * ‐ LOD, SPE—solid phase extraction, DSC‐MCAX—mixed‐mode sorbent, DMH4PT—dimethyltetrahydropterin, Isolute ENV+—hydroxylated polystyrene‐divinylbenzene copolymer.

It is becoming increasingly recognized that different oxidation states of the same pterin have distinct biological functions and clinical applications as disease biomarkers. A large number of oxidative pretreatments have been developed for their quantification in biological matrices. However, these pretreatments have proven problematic because the oxidation mechanisms are inefficient, complex and concentration‐dependent. On the other hand, the determination of pterins in their native oxidation states is complicated by their varying solubility, hydrophobicity, fluorescence, susceptibility to autooxidation, ionization efficiency, and molecular mass. However, the trend toward native pterin detection is gaining importance for expanding clinical applications [68].

Since fluorimetric detection, such as LIF, is limited by the weak intrinsic fluorescence of reduced pterins [44, 94], detection with mass spectrometry may be more suitable for determining their native state. Therefore, the combination of mass spectrometry with CE (CE–MS) and HPLC (HPLC–MS) represents a promising research direction. Finally, detection using high‐resolution mass spectrometry or tandem mass spectrometry offers several advantages over conventional mass spectrometers, including increased selectivity, sensitivity, and the ability to distinguish isobaric compounds such as 6,7‐DMP and 6‐formylpterin (6‐FP) (both *m*/*z* 192.1).

The combination of these techniques with the HILIC separation mode is becoming the predominant approach. The HILIC mode utilizes mobile phases with a high content of organic modifiers, which is advantageous for the ionization of pterins by ESI, particularly in the positive mode. This contrasts with RP chromatography, where achieving the retention of polar to highly polar pterins requires the use of a mobile phase with high water content approaching 100% which can generate irreproducible retention times, shorten the lifespan of analytical columns, and significantly reduce MS detection sensitivity. Another option for increasing the retention of polar pterins in RP is to add ion‐pairing reagents to the mobile phase composition; however, this can influence the effect of the matrix. Furthermore, fewer polar interferents (such as phospholipids) are eluted earlier in the HILIC system than in the RP system, thereby simplifying and accelerating the overall analysis. The robustness of these methods, combined with the detection of native pterins, leads to enhanced multiplexing capabilities, enabling the simultaneous quantification of a larger number of pterin derivatives—a field referred to as quantitative “pterinomics” [68]. Ultimately, these advances may enable system‐level analyses of pterin metabolism, allowing researchers to investigate entire pterin networks rather than individual compounds and thereby providing a more comprehensive understanding of their biological functions and diagnostic potential.

## 5 Conclusions

In conclusion, the analysis of pterins in biological samples encompasses a diverse array of methods, each tailored to address specific needs related to the type and stability of the samples under consideration. Alternative approaches, ranging from chemical oxidation to UV photoirradiation, now permit the limits of traditional methods to be overcome. Sample preparation strategies, such as the addition of stabilizing agents and solid‐phase extraction in particular have taken on a new importance for the reliability and accuracy of pterin analysis. Various separation and detection methods, like GC, CE, and LC, offer versatile options with varying degrees of sensitivity and selectivity. Mass spectrometry techniques, such as quadrupole tandem mass spectrometry and HPLC–Q/TOF‐MS, provide advanced possibilities for accurate identification and quantification of different forms of pterins. Application‐specific considerations, such as the choice of the biological matrix, further highlight the adaptability of these methods to meet diverse research needs. The continuous refinement and integration of analytical techniques will contribute to a better understanding of pterin biology and their potential as clinical biomarkers.

## Author Contributions

**Jindřich Brejcha**: conceptualization, writing – original draft preparation, writing – review and editing. **Zuzana Bosakova**: conceptualization, writing – original draft preparation, writing – review and editing, project administration, funding acquisition.

## Conflicts of Interest

The authors declare no conflicts of interest.

## Data Availability

Data sharing not applicable to this article as no datasets were generated or analyzed during the current study.
